# The Book World of Medicine and Science

**Published:** 1896-02-01

**Authors:** 


### THE BOOK WORLD OF MEDICINE AND SCIENCE.

SCIENCE PROGRESS. (The Scientific Press, Limited, 428, Strand, London, W.C.)

The February number of "Science Progress" will contain several interesting articles, amongst them "Emancipation from Scientific Materialism," by Professor W. Ostwald, of Leipsig; "Paulow's Researches on the Physiology of Secretion," by E. Starling, M.D.; and "The Suprarenal Capsules," by W. D. Halliburton, M.D., F.R.S.

HYGIENE. By J. LANE NOTTER, M.A., M.D., and H. R. FIRTH, F.R.C.S. (London: Longmans, Green, and Co. 1895. 3s. 6d.)

This is a nice readable book on the subject of hygiene, and will probably be much used by students, as it seems to contain in comparatively small compass a general sketch of most of the points a knowledge of which is now considered necessary on the part of the educated medical man. It is stated in the preface that the work is not to be regarded as a substitute for the regular and more advanced books which discuss the subject of hygiene in its many bearings, but rather as an introductory manual for the use of junior students, and this purpose it seems well calculated to serve. Its contents are well arranged, it is very well printed, and the different subjects dealt with are well indicated by headlines of darker type, so that it is easy to find one's way about. It is a pity that the authors have not adopted the metric system throughout the book, both in regard to weights, measures, temperatures, and pressures. There seems no excuse for the mixture of the different systems which we find in this book. The weariness to the reader is extreme, and it is impossible to see what object is served by it. For example, in the chapter on water both systems are used side by side for page after page, but when the latent heat of water comes to be spoken of it is described as 80 thermal units, no mention being made of the thermal unit on the other scale. Then we are told that rain "takes up from the atmosphere about 25 c.c. of gases per litre," and further down in the same paragraph that the average solid impurity in rain-water "is 2·8 grains in ten gallons." The qualitative examination of water, as regards lime and chlorine, is given in grains and gallons, but as regards nitrates, nitrites, &c., in c.c.s and litres; and then, when we come to quantitative work, the whole thing happily is given in the metric scale. But surely a student who can understand the metric system in quantitative analysis could understand it if used systematically throughout the book. Here and there we come upon lapses in regard to physics which we fear must be somewhat embarrassing to the student. For instance, under disinfection we find the following sentence, which surely conveys a wrong impression as to the meaning of the term "superheated" as applied to steam: "Hot air moistened with steam is somewhat better than hot dry air, but much inferior both in penetrating and germ-killing power to steam, especially steam which is superheated by being generated under pressure"; and further on, in regard to the action of the Washington Lyon apparatus, the same misunderstanding as to the meaning of superheating again crops up. It would be easy to lay hold of other passages in which, apparently from a want of crisp conceptions on the part of the authors, errors have crept in which must be puzzling to readers. For example, after stating that the respirations of those living in hot countries are so much lessened in frequency that the entire respiratory function is reduced by about 18½ per cent., it is said: "Much of this lessened respiratory function in hot climates is said to be due to the fact that with a high temperature the quantity of oxygen present in the air is diminished." Surely this does not properly explain the authors' meaning. These are instances sufficient to show that the book requires a careful revision before it can be considered absolutely reliable.

CRIMINAL SOCIOLOGY. By ENRICO FERRI, Professor of Criminal Law, Deputy in the Italian Parliament, &c. Criminology Series; edited by W. Douglas Morrison. (London: T. Fisher Unwin, 1895. Price 6s.)

Professor Ferri's "Criminal Sociology" comes appropriately after Lambroso's "Female Offender," in the admirable Criminology Series which Mr. Fisher Unwin is giving to the English public. This volume is perhaps less strictly scientific than its predecessor, and decidedly less likely to raise the suspicion that whenever we depart from the ordinary lines of beauty we show indications of a criminal type. Professor Ferri depends less on criminal anthropometry, and bases his study of criminal characteristics on wider grounds, while he is at one with his co-worker in regarding physical anomalies as being almost certain to be found in greater or less number in every criminal. He looks upon the criminal as a product of his heredity as well as of his environment, and therefore not a person likely to be redeemed from evil courses by human effort. He is anxious, however, that every criminal trial should be looked upon as a piece of psychological diagnosis, in which the mental and moral state of the criminal should be considered as of more importance than the crime, and for this reason prefers the trained intelligence of judges to the impulsive sympathies and prejudices of juries. We think, however, that in this he presupposes an amount of medical as well as legal knowledge in the judge which he does not often possess. Still there is no doubt that juries are often unduly influenced by circumstances which have nothing to do with the main point at issue in public prosecutions, namely, whether kindness or severity to the criminal is best for him and for the public against whom he has sinned. But since in England we are pledged to the jury system it is all the more desirable that handbooks such as these of the Criminology Series should be circulated as widely as possible in order to guide public intelligence how to look at both the evildoer and his deeds.

